# Possible poor prognosis in younger‐onset Crohn's disease‐associated anorectal cancer: A subanalysis of the Nationwide Japanese study

**DOI:** 10.1002/ags3.12773

**Published:** 2024-01-27

**Authors:** Yoshiki Okita, Yuji Toiyama, Hiroki Ikeuchi, Motoi Uchino, Kitaro Futami, Kinya Okamoto, Tatsuki Noguchi, Kenichi Sugihara, Soichiro Ishihara, Yoichi Ajioka, from the Study Group for Inflammatory Bowel Disease Associated Intestinal Cancers by the Japanese Society for Cancer of the Colon and Rectum

**Affiliations:** ^1^ Department of Gastrointestinal and Pediatric Surgery Mie University Graduate School of Medicine Tsu Japan; ^2^ Department of Gastroenterological Surgery, Division of Inflammatory Bowel Disease Hyogo Medical University Nishinomiya Japan; ^3^ Department of Surgery Fukuoka University Chikushi Hospital Chikushino Japan; ^4^ Department of Coloproctology Tokyo Yamate Medical Center Tokyo Japan; ^5^ Department of Surgical Oncology The University of Tokyo Tokyo Japan; ^6^ Tokyo Medical and Dental University Tokyo Japan; ^7^ Division of Molecular and Diagnostic Pathology, Graduate School of Medical and Dental Sciences Niigata University Niigata Japan

**Keywords:** anorectal cancer, Crohn's disease, surveillance

## Abstract

**Background and aims:**

Crohn's disease (CD)‐associated intestinal cancers are characterized by their high incidence, particularly at the anorectal site in the Japanese population. Accumulating evidence revealed that younger‐onset sporadic colorectal cancer may exhibit unique biological features. To the best of our knowledge, few previous articles reported clinicopathological features in patients with CD‐associated anorectal cancer (CDAAC). Therefore, we aimed to clarify the relationship between the younger onset of cancer and clinicopathological characteristics and prognosis, and the efficacy of cancer surveillance in patients with CDAAC.

**Methods:**

CD patients who had been diagnosed with intestinal cancers from 1983 to 2020 were collected from 39 Japanese institutions in this study. Of 316 patients with CD‐associated intestinal cancers, we analyzed 211 patients with CDAAC. We divided the patients into two groups according to the median age at cancer diagnosis (45 years old).

**Results:**

Younger‐onset CDAAC (YO‐CDAAC) patients were significantly more likely to have a poor outcome than those with older‐onset CDAAC (OO‐CDAAC) in terms of both disease‐free survival (DFS) (*p* = 0.0014) and overall survival (OS) (*p* = 0.023). Multivariate analysis showed that age under 45 years old at diagnosis of cancer was one of the independent factors for poor DFS and OS (hazard ratios: 2.15, 95% confidence interval: 1.09–4.26, *p* = 0.028, hazard ratios: 1.95, 95% confidence interval: 1.05–3.60, *p* = 0.033, respectively). Patients detected via surveillance showed significantly better DFS and OS rates than symptomatic patients in YO‐CDAAC (*p* = 0.012 and 0.0031, respectively).

**Conclusions:**

YO‐CDAAC may have a poorer prognosis compared with OO‐CDAAC. Surveillance could be important to improve cancer prognosis, especially in young CD patients with anorectal disease.

## Graphical abstract

Younger‐onset Crohn's disease‐associated anorectal cancer may have a poorer prognosis compared with older‐onset Crohn's disease‐associated anorectal cancer. Surveillance could be important to improve cancer prognosis, especially in young Crohn's disease patients with anorectal disease.

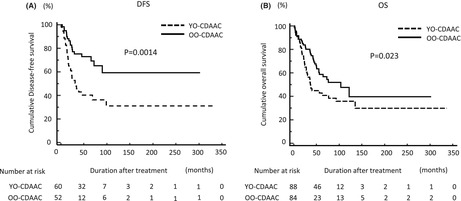

## 1 INTRODUCTION

Ulcerative colitis (UC) is a well‐defined risk factor for colorectal cancer, and the duration and extent of this disease have been identified as important risk factors.^1^ Although there was believed to be little incidence of Crohn's disease (CD)‐associated cancer, an increased rate of intestinal cancer has been assigned to CD in various studies.^2^, ^3^, ^4^, ^5^ However, a limited number of CD‐associated cancers have been analyzed and reported thus far.

CD‐associated colorectal cancers are characterized by a high incidence in the distal colon, particularly at the anorectal site, in the Japanese population.^6^, ^7^ These findings indicate that the characteristics of cancer may differ on the basis of geography or ethnic background.^6^

Several studies have reported that CD‐associated colorectal cancers are likely to have a poorer prognosis compared with sporadic colorectal cancer (SCRC).^6^, ^7^, ^8^ However, prognostic factors in patients with CD‐associated anorectal cancer (CDAAC) have not been reported. Moreover, the efficacy of surveillance for early detection of CDAAC has not been demonstrated.

Younger‐onset SCRC, which is often defined as occurring in patients aged under 50 years, has been increasing worldwide.^9^, ^10^ Younger‐onset SCRC is often characterized by a more advanced stage, poorer cell differentiation, left‐sided location, and a proportion of cancer family syndromes.^11^, ^12^ This suggests that younger‐onset SCRC may exhibit unique biological features and a potentially different prognosis when compared with SCRC diagnosed at an older age.^12^

In this study, we hypothesized that there are some differences between younger‐onset CD‐associated anorectal cancer (YO‐CDAAC) and older‐onset CD‐associated anorectal cancer (OO‐CDAAC) regarding clinicopathological features and cancer prognosis. Therefore, we examined clinicopathological characteristics and cancer prognosis in patients with CDAAC, and the efficacy of cancer surveillance in patients with CDAAC by using a large nationwide database from the Japanese Society for Cancer of the Colon and Rectum (JSCCR).^13^

## 2 METHODS

Patients' data were collected from 39 institutions, including from surgery and gastroenterology departments, that are members of JSCCR. CD patients who had been diagnosed with intestinal cancers from 1983 to 2020 were collected in this study. Of these, CD patients who had rectal and/or anal cancer were extracted and analyzed. The patients' data were retrospectively collected from medical records at each institution and then sent to the Department of Surgical Oncology at the University of Tokyo for further analysis.

Patients' characteristics, namely sex, age at CD onset, disease duration, age at diagnosis of cancer, disease behavior, presence of colonic disease, presence of anal ulcer, presence of perianal fistula, use of steroids, use of biologics therapy, use of immunomodulators, use of adjuvant chemotherapy, surgical factors including surgical approach, indication and curability, histopathological findings in resected specimens including histology, pathological T factor, lymphatic invasion, venous invasion, node involvement, distant metastasis, recurrence type, and long‐term oncological outcomes were collected for this study.

Among 316 patients with CD‐associated intestinal cancers, 211 (73%) had anorectal cancer. The median age at diagnosis of CD was 24 years old and the median disease duration was 244.5 months in patients with anorectal cancer. The median age at cancer diagnosis was 45 years old in patients with anorectal cancer. The study population was divided into two groups: age at cancer diagnosis under 45 years old (YO‐CDAAC group) and age at cancer diagnosis over 45 years old (OO‐CDAAC group). In addition, treatment guidelines for CD anal lesions were established by the Japan Ministry of Health, Labor, and Welfare in 2008.^14^ Therefore, we added the period of treatment divided into 1983–2008 and 2009–2020 as a factor related with prognosis.

### 2.1 Ethics

This multicenter retrospective study was approved by the ethics committee of the University of Tokyo [2019220NI‐(2)], the ethics committees of each institution if necessary, and the ethics committee of JSCCR. The requirement for written informed consent from patients for participation in this study was waived due to the retrospective design of the study.

### 2.2 Statistical analysis

Statistical analyses were performed using JMP Pro 16 (SAS Institute Inc, Cary, NC, USA). Comparisons of the clinicopathological characteristics between groups were evaluated using Pearson's chi‐squared test or Fisher's exact test for categorical variables, as appropriate. Continuous variables were shown as the means with standard deviations and were compared with Student's *t* test results. Survival analysis was performed using the Kaplan–Meier method and was compared using the log‐rank test. Cox proportional hazards models were used to estimate hazard ratios (HR) for recurrence and death. Variables with *p* < 0.05 in the univariate analysis were selected for multivariate analysis using the Cox proportional hazards regression model. Disease‐free survival (DFS) was measured from the date the patient underwent surgery to the date of disease recurrence, death from any cause (i.e., cancer‐unrelated deaths were not censored), or until the last known follow‐up in patients who were still alive. DFS was analyzed only for pathological stage 0–III patients who underwent curative resection (CurA). Overall survival (OS) was measured from the date the patient underwent surgery until the date of death resulting from any cause, or until the last known follow‐up in patients who were still alive. The patients without information of prognosis and with follow‐up period less than 12 months were excluded in DFS and OS analysis.

## 3 RESULTS

### 3.1 Clinicopathological characteristics of CD‐associated anorectal cancer

The clinicopathological characteristics of overall patients with CDAAC in this study are shown in Table 1. The median follow‐up period was 33.6 (range, 0.4–337) months in 211 CD patients. The current study noted a 5‐year OS rate of 55.3%.

**TABLE 1:** Clinicopathological characteristics of patients with CD‐associated anorectal cancer.

Sex	Male/female	141 (66.8%)/70 (33.2%)
Age at diagnosis of Crohn's disease	Years, mean ± SD	26.9 ± 10.9
Disease duration	Months mean ± SD	253.3 ± 117
Age at diagnosis of cancer	Years, mean ± SD	45.4 ± 10.5
Stricturing	Presence/Absence	115 (56.7%)/88 (43.3%)
Penetrating	Presence/Absence	83 (40.5%)/122 (59.5%)
Colonic disease	Presence/Absence	186 (88.2%)/25 (11.8%)
Anal ulcer	Presence/Absence	15 (8.3%)/165 (91.7%)
Perianal fistula	Presence/Absence	152 (73.4%)/55 (26.6%)
Preoperative medication		
Use of steroid	Presence/Absence	32 (21.1%)/119 (78.8)
Use of biologics	Presence/Absence	57 (27.0%)/154 (73.0%)
Use of immunomodulator	Presence/Absence	19 (9.0%)/192 (91.0)
Indication	Malignancy/Benign	166 (86.4%)/37 (13.6%)
Approach	Open/Laparoscopy, Robot	172 (83.7%)/27 (16.3%)
Curability	CurA/Others	133 (70%)/57 (30.0%)
Dominant histology type	Wel (tub1)/Mod (tub2)/Por/Muc/Sig/SCC	43 (22.8%)/24 (12.7%)/4 (2.1%)/100 (52.9%)/13 (6.9%)/5 (2.6%)
TNM classification		
T stage	is/1a,1b/2/3/4a/4b/X	16 (8.5%)/10 (5.3%)/29 (15.3%)/68 (36.0%)/18 (9.5%)/49 (25.9%)/5 (2.6%)
Node involvement	Node involvement	59 (32.2%)/124 (67.8%)
Distant metastasis	Distant metastasis	21 (10.4%)/180 (89.6%)
TNM stage	0/1/2a/2b/2c/3a/3b/3c/4/X	17 (9.0%)/28 (14.8%)/47 (24.9%)/7 (3.7%)/21 (11.1%)/5 (2.6%)/17 (9.0%)/26 (13.8%)/17 (9.0%)/5 (2.6%)

Abbreviations: Mod(tub2), moderately differentiated adenocarcinoma; Muc, mucinous carcinoma; Por, poorly differentiated adenocarcinoma; SCC, squamous cell carcinoma; Sig, signet‐ring cell carcinoma; Wel(tub1), well‐differentiated adenocarcinoma.

### 3.2 Comparison of the clinicopathological characteristics between younger‐onset and older‐onset CD‐associated anorectal cancer

One patient had no information about age at diagnosis of cancer. CDAAC patients comprised 103 YO‐CDAAC patients and 107 OO‐CDAAC patients. We compared the clinicopathological characteristics between YO‐CDAAC and OO‐CDAAC (Table 2). Age at diagnosis of CD in YO‐CDAAC was significantly younger compared with OO‐CDAAC (24.1 ± 7.7 vs. 30.5 ± 12.0, *p* = 0.0009). Disease duration in YO‐CDAAC was significantly shorter compared with OO‐CDAAC (182 ± 78 vs. 302 ± 116, *p* < 0.0001). The proportion of patients with well‐ or moderately differentiated adenocarcinoma (Tub1 or Tub2) was significantly lower in YO‐CDAAC (20.4% vs. 45.5%, *p* = 0.0002). Patients with YO‐CDAAC were more frequently diagnosed with advanced tumor stage (beyond T3) (79.1% vs. 63.3%, *p* = 0.024), and advanced TNM stage (beyond pathological stage II) (84.6% vs. 66.7%, *p* = 0.0059), but there were no significant differences in lymphatic invasion (44.6% vs. 36.8%, *p* = 0.36), and venous invasion (43.4% vs. 31.6%, *p* = 0.15) between YO‐CDAAC and OO‐CDAAC. Patients with YO‐CDAAC tended to be more frequently diagnosed with node involvement (39.8% vs. 25.5%, *p* = 0.057). There were no differences in local (56.3% vs. 73.7%, *p* = 0.25), lymph node recurrence (12.9% vs. 5.0%, *p* = 0.64), liver (3.2% vs. 10.5%, *p* = 0.55), lung (15.6% vs. 26.3%, *p* = 0.47), or peritoneum (12.9% vs. 15.0%, *p* = 1.00) recurrence between YO‐CDAAC and OO‐CDAAC in patients with pathological stage 0–III patients who underwent curative resection.

**TABLE 2:** Comparison of the clinicopathological characteristics between younger‐onset CD‐associated anorectal cancer and older‐onset CD‐associated anorectal cancer.

		YO‐CDAAC	OO‐CDAAC	*p*‐Value
Sex	Male/female	69 (67.0%)/34 (33.0%)	71 (66.4%)/36 (33.6%)	1.00
The year of cancer diagnosis	≤2008, ≥2009	29 (28.2%)/74 (71.8%)	20 (18.7%)/87 (81.3%)	0.14
Crohn's disease‐related factor				
Age at diagnosis of Crohn's disease	Years, mean ± SD	24.1 ± 7.7	30.5 ± 12.0	0.0009
Disease duration	Months, mean ± SD	182 ± 78	302 ± 116	<0.0001
Stricturing	Presence/Absence	58 (56.9%)/44 (43.1%)	57 (56.4%)/44 (43.6%)	0.89
Penetrating	Presence/Absence	44 (43.1%)/58 (56.9%)	39 (37.9%)/64 (62.1%)	0.48
Colonic disease	Presence/Absence	93 (90.3)/10 (9.7%)	92 (86.0%)/15 (14.0%)	0.40
Anal ulcer	Presence/Absence	6 (6.8%)/82 (93.2%)	9 (9.8%)/83 (90.2%)	0.59
Perianal fistula	Presence/Absence	74 (72.5%)/28 (27.5%)	78 (74.3%)/27 (25.7%)	0.88
Diagnostic procedure	Surveillance/Symptom	29 (32.2%)/61 (67.8%)	34 (34.7%)/64 (65.3%)	0.76
Surgical factor				
Indication	Malignancy/Benign	86 (86.0%)/14 (14.0%)	80 (77.7%)/23 (22.3%)	0.15
Approach	Open/Laparoscopy, Robot	82 (83.7%)/16 (16.3%)	90 (89.1%)/11 (10.9%)	0.30
Curability	CurA/Others	64 (69.6%)/28 (30.4%)	69 (70.4%)/29 (29.6%)	1.00
Adjuvant chemotherapy	Presence/Absence	47 (49.0%)/49 (51.0%)	47 (46.5%)/54 (53.5%)	0.78
Histological factor				
Dominant histology type	Tub/Others	20 (20.4%)/78 (79.6%)	46 (45.5%)/55 (54.5%)	0.0002
T stage	T 0–2/T3–4	19 (20.9%)/72 (79.1%)	36 (36.7%)/62 (63.3%)	0.024
Lymphatic invasion	Presence/Absence	37 (44.6%)/46 (55.4%)	35 (36.8%)/60 (63.2%)	0.36
Venous invasion	Presence/Absence	36 (43.4%)/47 (56.6%)	30 (31.6%)/65 (68.4%)	0.15
Node involvement	Presence/Absence	35 (39.8%)/53 (60.2%)	24 (25.5%)/70 (74.5%)	0.057
TNM stage	0–I/II–IV	14 (15.4%)/77 (84.6%)	31 (33.3%)/62 (66.7%)	0.0059
Recurrence type				
Local	Presence/Absence	18 (56.3%)/14 (43.8%)	14 (26.3%)/5 (73.7%)	0.25
Lymph node	Presence/Absence	4 (12.9%)/27 (87.1%)	1 (5.0%)/19 (95.0%)	0.64
Liver	Presence/Absence	1 (3.2%)/30 (96.8%)	2 (10.5%)/17 (89.5%)	0.55
Lung	Presence/Absence	5 (15.6%)/27 (84.4%)	5 (26.3%)/14 (73.7%)	0.47
Peritoneum	Presence/Absence	4 (12.9%)/27 (87.1%)	3 (15.0%)/17 (85.0%)	1.00

Abbreviations: OO‐CDAAC, older‐onset Crohn's disease‐associated anorectal cancer, tub, well+ moderately differentiated adenocarcinoma; YO‐CDAAC, younger‐onset Crohn's disease‐associated anorectal cancer.

### 3.3 Younger‐onset CD‐associated anorectal cancer showed a poor prognosis

**FIGURE 1:**
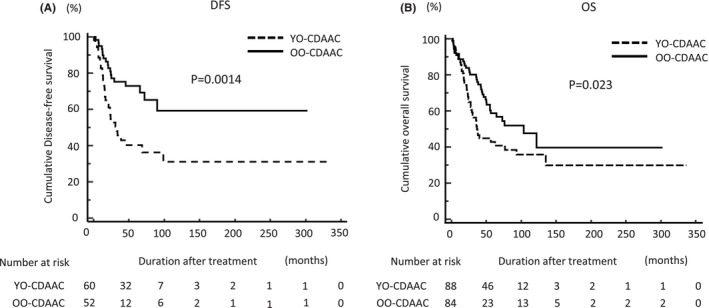
Prognostic impact of age at diagnosis of cancer on DFS and OS in patients with CDAAC. YO‐CDAAC patients were significantly more likely to have a poor outcome than OO‐CDAAC patients in terms of both (A) DFS and (B) OS. DFS, disease‐free survival; OO‐CDAAC, older‐onset Crohn's disease‐associated anorectal cancer; OS, overall survival; YO‐CDACC, younger‐onset Crohn's disease‐associated anorectal cancer.

We investigated the prognostic impact of age at diagnosis of cancer on DFS and OS in patients with CDAAC. According to Kaplan–Meier survival curves subdivided by age at diagnosis of cancer, YO‐CDAAC patients had a significantly poorer prognosis than those with OO‐CDCARC in terms of DFS (log‐rank test, *p* = 0.0014; Figure 1A). YO‐CDAAC patients also had a significantly poorer prognosis than those with OO‐CDCARC in terms of OS (log‐rank test, *p* = 0.023; Figure 1B).

### 3.4 Age at diagnosis of cancer was an independent factor for DFS and OS

To clarify the potential utility of age at diagnosis of cancer as a prognostic predictor for recurrence and survival, we conducted a Cox proportional hazards regression analysis for DFS and OS in patients with CDAAC (Tables 3 and 4).

**TABLE 3:** Uni‐ and multivariate analyses of predictors of cancer recurrence in patients with Crohn's disease‐associated anorectal cancer.

		Univariate analysis			Multivariate analysis		
		HR	95% CI	*p*‐Value	HR	95% CI	*p*‐Value
Sex	Male	0.71	0.40–1.26	0.24			
Age at diagnosis of Crohn's disease	<24 years old	1.25	0.71–2.21	0.44			
Disease duration	<244.5 months	1.24	0.70–2.20	0.46			
Age at diagnosis of cancer	<45 years old	2.60	1.45–4.66	0.0013	2.15	1.09–4.26	0.028
The year of cancer diagnosis	≤2008	1.89	1.02–3.49	0.043	1.07	0.47–2.45	0.88
Stricturing	Presence	1.07	0.61–1.89	0.81			
Penetrating	Presence	2.17	1.23–3.84	0.0078	1.18	0.60–2.32	0.62
Colonic disease	Presence	0.66	0.31–1.41	0.29			
Anal ulcer	Presence	0.97	0.38–2.46	0.97			
Perianal fistula	Presence	1.89	0.94–3.80	0.072			
Use of steroid	Yes	1.51	0.77–2.97	0.23			
Use of biologics	Yes	0.64	0.31–1.33	0.23			
Use of immunomodulator	Yes	0.44	0.11–1.83	0.26			
Dominant histology type	Tub	0.57	0.30–1.10	0.096			
T factor	T3/4	4.85	2.17–10.85	0.0001	2.59	1.08–6.23	0.033
Lymphatic invasion	Presence	2.14	1.20–3.82	0.010	1.30	0.55–3.09	0.55
Venous invasion	Presence	2.09	1.17–3.76	0.013	0.84	0.34–2.08	0.71
Node involvement	Presence	4.33	2.42–7.73	<0.0001	1.97	0.90–4.33	0.091
Adjuvant chemotherapy	Presence	4.96	2.47–9.95	<0.0001	2.50	1.03–6.06	0.043
Surveillance	Presence	0.47	0.25–0.89	0.020	0.42	0.19–0.94	0.035

Abbreviation: tub, well+ moderately differentiated adenocarcinoma.

**TABLE 4:** Uni‐ and multivariate analyses of predictors of mortality in patients with Crohn's disease‐associated anorectal cancer.

		Univariate analysis			Multivariate analysis		
		HR	95%CI	*p*‐Value	HR	95%CI	*p*‐Value
Sex	Male	0.81	0.52–1.26	0.35			
Age at diagnosis of Crohn's disease	<24 years old	1.22	0.78–1.88	0.38			
Disease duration	<244.5 months	0.74	0.47–1.17	0.20			
Age at diagnosis of cancer	<45 years old	1.65	1.07–2.56	0.024	1.95	1.05–3.60	0.033
The year of cancer diagnosis	≤2008	1.88	1.20–2.96	0.0061	1.82	0.98–3.39	0.059
Stricturing	Presence	1.23	0.79–1.91	0.37			
Penetrating	Presence	1.62	1.04–2.51	0.033	1.01	0.53–1.92	0.98
Colonic disease	Presence	1.01	0.56–1.83	0.97			
Anal ulcer	Presence	1.68	0.90–3.14	0.11			
Perianal fistula	Presence	1.84	1.03–3.29	0.038	1.23	0.57–2.65	0.59
Use of steroid	Yes	1.03	0.55–1.91	0.93			
Use of biologics	Yes	0.58	0.32–1.04	0.068			
Use of immunomodulator	Yes	0.43	0.14–1.36	0.15			
Dominant histology type	Tub	0.50	0.30–0.84	0.0091	0.63	0.32–1.22	0.17
T factor	T3/4	6.22	2.84–13.59	<0.0001	2.97	1.26–6.97	0.012
Lymphatic invasion	Presence	2.58	1.58–4.23	0.0002	1.51	0.63–3.59	0.35
Venous invasion	Presence	2.80	1.72–4.56	<0.0001	1.05	0.43–2.54	0.91
Node involvement	Presence	4.10	2.54–6.60	<0.0001	1.50	0.75–2.99	0.25
Distant metastasis	Presence	11.43	6.50–20.09	<0.0001	5.24	1.88–14.60	0.0015
Adjuvant chemotherapy	Presence	1.90	1.20–3.03	0.0064	1.94	0.95–3.97	0.070
Surveillance	Presence	0.39	0.22–0.68	0.0010	0.42	0.18–0.94	0.036

Abbreviation: tub, well+ moderately differentiated adenocarcinoma.

Multivariate analysis showed that age under 45 at diagnosis of cancer [HR: 2.15, 95% confidence interval (CI): 1.09–4.26, *p* = 0.028], T factor (T3/4) (HR: 2.59, 95% CI: 1.08–6.23, *p* = 0.033), adjuvant chemotherapy (HR: 2.50, 95% CI: 1.03–6.06, *p* = 0.043), and absence of surveillance (HR: 0.42, 95% CI: 0.19–0.94, *p* = 0.035) were independent factors for poor DFS in patients with CDAAC. In terms of OS, age under 45 at diagnosis of cancer [HR: 1.95, 95% confidence interval (CI): 1.05–3.60, *p* = 0.033], T factor (T3/4) (HR: 2.97, 95% CI: 1.26–6.97, *p* = 0.012), distant metastasis (HR: 5.24, 95% CI: 1.88–14.60, *p* = 0.0015), and absence of surveillance (HR: 0.42, 95% CI: 0.18–0.94, *p* = 0.036) were independent factors for poor OS in patients with CDAAC.

### 3.5 Cancer detection via surveillance was associated with better outcome in patients with younger‐onset CD‐associated anorectal cancer

Only 29.9% of the CD‐associated cancers were diagnosed by surveillance, whereas 59.2% of cancers were diagnosed as a result of workups for symptoms in overall patients with CDAAC. Among the surveillance modalities for CD cancers, endoscopic examination was the most frequent (76.2%), followed by trans‐anal biopsy in 15.9% of cases.

**FIGURE 2:**
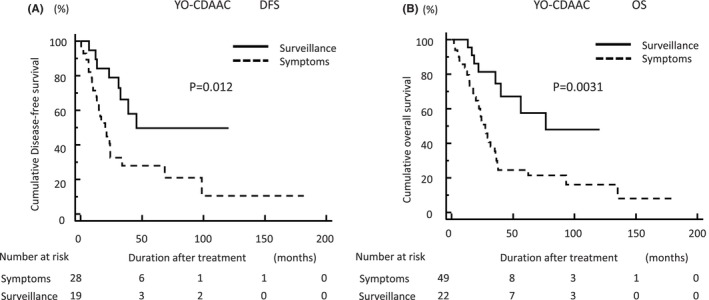
Comparison of DFS and OS in YO‐CDAAC for cancer detection via surveillance and in symptomatic cases. Cancers detected via surveillance showed significantly better (A) DFS and (B) OS rates than symptomatic cases. DFS, disease‐free survival; OO‐CDAAC, older‐onset Crohn's disease‐associated anorectal cancer; OS, overall survival; YO‐CDACC, younger‐onset Crohn's disease‐associated anorectal cancer.

**FIGURE 3:**
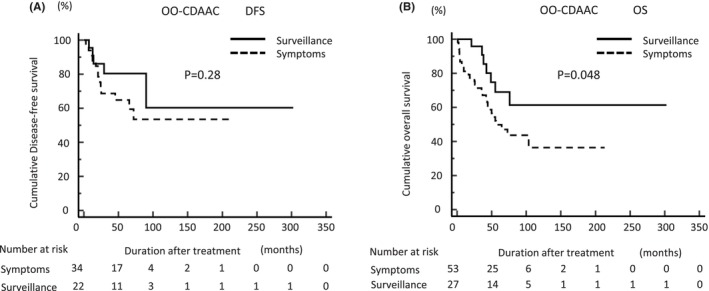
Comparison of DFS and OS in OO‐CDAAC for cancer detection via surveillance and in symptomatic cases. Cancers detected via surveillance did not show better (A) DFS but showed better (B) OS rates than symptomatic cases. DFS, disease‐free survival; OO‐CDAAC, older‐onset Crohn's disease‐associated anorectal cancer; OS, overall survival; YO‐CDACC, younger‐onset Crohn's disease‐associated anorectal cancer.

To clarify the potential efficacy of surveillance for recurrence and survival in CDAAC, we compared DFS and OS of YO‐CDAAC or OO‐CDAAC following cancer detection via surveillance and in symptomatic cases. Cancers detected via surveillance showed significantly better DFS and OS than symptomatic cases in YO‐CDAAC patients (*p* = 0.012 and *p* = 0.0031, respectively: Figure 2A,B). On the other hand, cancers detected via surveillance didn't show significantly better DFS than symptomatic cases but showed significantly better OS than symptomatic cases in OO‐CDAAC patients (*p* = 0.28 and *p* = 0.048, respectively: Figure 3A,B).

**FIGURE 4:**
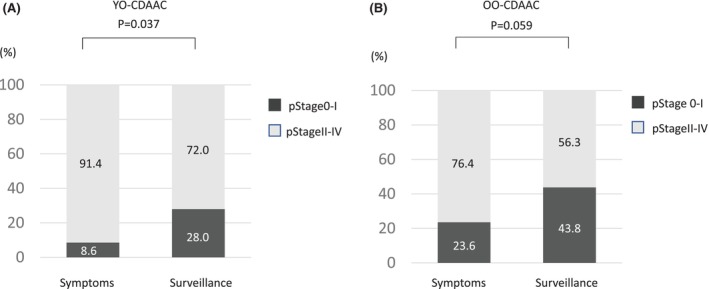
Comparison of TNM stage in YO‐CDAAC and OO‐CDAAC for cancer detection via surveillance and in symptomatic cases. (A) In patients with YO‐CDAAC, a significantly lower proportion of stage 0–I cancers were detected as a result of workups for symptoms compared to surveillance. (B) In patients with OO‐CDAAC, a lower proportion of stage 0–I cancers tended to be detected as a result of workups for symptoms compared to surveillance. OO‐CDAAC, older‐onset Crohn's disease‐associated anorectal cancer; YO‐CDACC, younger‐onset Crohn's disease‐associated anorectal cancer.

In patients with YO‐CDAAC, a significantly less proportion of stage 0–I cancers were detected as a result of workups for symptoms compared to surveillance (8.6% vs. 28.0%, *p* = 0.037) (Figure 4A). In patients with OO‐CDAAC, a lower proportion of stage 0–I cancers tended to be detected as a result of workups for symptoms compared to surveillance (23.6% vs. 43.8%, *p* = 0.058) (Figure 4B).

### 3.6 Surveillance was an independent factor for DFS in patients under 45 years old, but not over 45 years old at diagnosis of cancer

To clarify the potential utility of surveillance for recurrence in YO‐CDAAC or OO‐CDAAC, we conducted a Cox proportional hazards regression analysis for DFS in patients with CDAAC divided by age at diagnosis of cancer (Tables S1 and S2). In patients under 45 years old, multivariate analysis showed that T factor (T3/4) (HR: 9.94, 95% CI: 1.69–58.37, *p* = 0.011) and absence of surveillance (HR: 0.28, 95% CI: 0.09–0.85, *p* = 0.025) were independent factors for poor DFS. In patients over 45 years old, multivariate analysis showed that node involvement (HR: 3.09, 95% CI: 1.14–8.37, *p* = 0.027) and adjuvant chemotherapy (HR: 4.56, 95% CI: 1.24–16.73, *p* = 0.022) were independent factors for poor DFS, but surveillance did not show significant association with DFS (HR: 0.71, 95% CI: 0.22–2.24, *p* = 0.56).

### 3.7 Surveillance was an independent factor for OS in patients both under 45 years old and over 45 years old at diagnosis of cancer

To clarify the potential utility of surveillance for mortality in YO‐CDAAC or OO‐CDAAC, we conducted a Cox proportional hazards regression analysis for OS in patients with CDAAC divided by age at diagnosis of cancer (Tables S3 and S4). In patients with age under 45, multivariate analysis showed that T factor (T3/4) (HR: 11.13, 95% CI: 1.41–87.97, *p* = 0.022), distant metastasis (HR:7.72, 95% CI: 1.74–34.30, *p* = 0.0072), and absence of surveillance (HR: 0.27, 95% CI: 0.097–0.73, *p* = 0.0097) were independent factors for poor OS. In patients aged over 45, univariate analysis show that absence of surveillance was prone to be associated with poor OS (HR: 0.44, 95% CI: 0.19–1.02, *p* = 0.055). Multivariate analysis showed that use of biologics (HR:0.060, 95% CI: 0.011–0.31, *p* = 0.0008), lymphatic invasion (HR:3.45, 95% CI: 1.05–11.27, *p* = 0.041), node involvement (HR:5.85, 95% CI: 1.84–18.59, *p* = 0.028), distant metastasis (HR:8.05, 95% CI: 1.64–39.47, *p* = 0.010), and absence of surveillance (HR: 0.12, 95% CI: 0.035–0.38, *p* = 0.0004) were independent factors for poor OS.

## 4 DISCUSSION

CD‐related cancers are known to vary widely regarding site of origin depending on ethnic background and geography. Higashi et al.^7^ demonstrated that CD‐associated cancer was most prevalent in the anal canal (51%) followed by the rectum (29%) using data collected from 16 institutional facilities in Japan. Uchino et al.^6^ also analyzed 54 studies including 208 patients with CD‐associated cancer by systematic review and meta‐analysis. In Uchino's report, CD‐associated cancer in the right side of the colon occurred more frequently in Western countries than in Asian countries.^6^ In contrast, a much higher incidence of anorectal cancer was observed in Asian countries than in Western countries.^6^ Examination under anesthesia with lower anorectal site biopsy should be conducted in addition to endoscopic surveillance for perineal CD for improving prognosis.^15^ Accordingly, we focused on CD‐associated cancer located at the anorectal site in this study.

Several reports have demonstrated that CDAAC has a poorer prognosis than SCRC. Uchino et al.^6^ demonstrated that the median survival in patients with CDAAC was 2.1 years, and advanced cancer greater than stage T3 occurred in 62.1%. Ogino et al.^16^ revealed that the 5‐year OS of CD‐associated rectal cancer was significantly worse than that of sporadic rectal cancer (51.61% vs. 68.58%; *p* < 0.001), whereas the 5‐year OS of CD‐associated colon cancer versus sporadic colon cancer was comparable. Other studies also reported that 5‐year survival rates were 44%–52%, indicating a poor prognosis in CDAAC.^7^, ^17^, ^18^, ^19^ The poor prognosis of CDAAC might be explained by the fact that most anorectal cancers are at an advanced stage at the time of diagnosis.

In addition, the histopathological characteristics of SCRC and CD‐associated cancer differ significantly. In fact, Higashi et al.^7^ showed that the proportion of mucinous carcinoma was 50%, well‐ and moderately differentiated was 42%, and poorly differentiated was 4% in CD‐associated cancer. Other studies have also demonstrated that mucinous carcinoma, poorly differentiated adenocarcinoma, or signet‐ring cell carcinoma occurred more frequently in CD‐associated cancer than SCRC.^15^, ^17^ Furthermore, squamous cell cancer (SCC) is a characteristic histopathological finding in CDAAC and is more frequent in Western countries.^6^ In addition, although perianal fistulizing CD is at risk of perianal fistula‐related SCC, the incidence rate of SCC is very low (<1%).^20^ This was also shown in our data, in five out of 211 patients (2.5%) with CDAAC.

Incidence rates of SCRC have remained stable or have declined in many high‐income countries. In contrast, younger‐onset SCRC has recently been increasing worldwide, although the causes of the rise in younger‐onset SCRC remain uncertain.^21^ A number of reports about the clinicopathological features and prognosis of young patients with SCRC have been published.^21^ However, these reports have no consistency in terms of prognostic associations of younger‐onset SCRC.^21^

To ascertain whether a poorer prognosis in patients with younger age at diagnosis of cancer is indeed a distinct characteristic of CDAAC, we conducted a comparison of DFS or OS between younger‐onset CD‐associated colon cancer and older‐onset CD‐associated colon cancer (Figure S1). According to Kaplan–Meier survival curves subdivided by age at diagnosis of cancer, there were no significant differences in both DFS and OS between them (log‐rank test, DFS; *p* = 0.95, OS; *p* = 0.68).

With this background, we focused on the relationship between age at diagnosis of cancer and its clinicopathological features and prognosis in patients with CDAAC. Patients with YO‐CDAAC were more frequently diagnosed with non‐differentiated adenocarcinoma, advanced tumor stage, node involvement, and advanced TNM stage. In addition, YO‐CDAAC patients showed a poor prognosis for both DFS and OS. Moreover, age at diagnosis of cancer was an independent predictive factor for poor DFS and OS.

Chen et al.^22^ showed that a greater proportion of patients younger than 50 years old were diagnosed with advanced‐stage (stage III–IV) tumors than older patients in a retrospective analysis of patients with SCRC. In the characteristics of patients with younger‐onset SCRC in their study, longer median time to diagnosis, symptom duration, and time of evaluation were significantly recognized.^22^ In addition, it was reported that lower awareness of cancer, lack of screening, an underappreciation of symptoms, and reluctance to take medical care may cause delayed diagnosis and advanced stage at diagnosis in younger‐onset SCRC patients.^23^ Consistent with the characteristic features of younger‐onset SCRC patients, patients with YO‐CDAAC were more frequently diagnosed with advanced TNM stage in our study. In addition, the data in this study did not distinguish between symptoms due to cancer and intestinal complications. Therefore, the difference in the percentage of Stage 0–I cancers in the surveillance group and the symptomatic group between YO‐CDACC and OO‐CDACC, as shown in Figure 4, might be attributed to the influence of early‐stage cancers that had been diagnosed by chance in specimens resected due to intestinal complications in the symptomatic group.

Adjuvant chemotherapy was identified as an independent factor for poor DFS analysis in overall patients and in patients over 45 years old at the time of cancer diagnosis. Adjuvant chemotherapy was not identified as an independent factor for poor DFS in patients under 45 years old and for OS in this study. Adjuvant chemotherapy is generally indicated based on the presence of node involvement. The data could potentially be influenced by confounding factors. Therefore, it's important to carefully analyze the variables involved and consider potential sources of bias.

Use of biologics was also identified as an independent factor for poor OS analysis in patients over 45 years old at the time of cancer diagnosis. In survival analysis, the use of biologics shows a significantly better OS compared to no use of biologics in OO‐CDAAC patients (*p* = 0.022, log‐rank test; data not shown). The effectiveness of biologics may improve the general condition and consequently lead to better OS. However, the precise underlying causes for this phenomenon remain undisclosed. Further studies investigating this association are needed.

We also demonstrated that surveillance showed significantly better DFS and OS rates than symptomatic cases among the YO‐CDAAC patients. However, surveillance did not show significantly better DFS, but show significantly better OS rates than symptomatic cases among the OO‐CDAAC patients. In patients with YO‐CDAAC, the symptomatic cases were correlated with a cancer diagnosis at a significantly more advanced stage compared to surveillance cases. These findings suggest the importance of surveillance especially for younger CD patients. Previous studies have suggested that surveillance can improve the prognosis of UC‐associated cancers by enabling diagnosis at earlier disease stages.^24^, ^25^ Noguchi et al.^13^ retrospectively analyzed a total of 1505 intestinal cancers in 1189 UC and 316 CD patients treated at 43 Japanese institutions. The study reported that cancers found by surveillance showed a significantly better OS rate than the symptomatic cases in the UC and CD patients. In CD, surveillance colonoscopy is also recommended by several guidelines because of the higher incidence of colorectal cancer compared with the general population.^26^, ^27^, ^28^ In these guidelines, surveillance colonoscopy is recommended at 8–10 years after the onset of CD, similar to UC. However, it is difficult to survey, including pathological analysis, for anorectal lesions of CD because of it being complicated by anorectal stricture, perianal abscess, and perianal fistula. Kotsafti et al.^29^ recommended that examination under anesthesia should be performed when findings such as a change in symptoms or simply long‐standing disease in the perineum are present. Therefore, because of the difficulty of undertaking surveillance to detect CDAAC, no previous studies except that of Hirano et al. examined whether surveillance could improve the prognosis of patients with CDAAC.^15^

There are several limitations of this study. First, this study was retrospective. We revealed the year of cancer diagnosis during 2009–2020 was significantly associated with better DFS and OS compared with 1980–2008 in univariate analysis. The results of this study might be affected by changes in medical and surgical approaches to CD due to a long study period. However, this study precisely examined and reported a relatively large number of CDAAC cases, and patient data were collected from a wide variety of centers, including those not specializing in IBD treatment, thus representing real‐world clinical practice. The second limitation was this study had some missing data due to the retrospective registration of data. However, percentages of the missing data for most of the variables were less than 10%, and the sensitivity analyses using the imputed data set revealed similar results. The third limitation was that the diagnoses of CD and CD‐associated cancers were made at each institution and not centralized. The fourth limitation was that consistent surveillance programs have not been performed for CDAAC. Data in this study did not include information regarding the surveillance method, such as the subject of the surveillance (disease duration, extent of disease), the interval of surveillance, and the procedure (use of colonoscopy or examination under anesthesia).

In conclusion, patients with YO‐CDAAC are more likely to be diagnosed at a later stage and show a lower frequency of well‐ or moderately differentiated adenocarcinoma and poor prognosis in CDAAC. Because our study shows that surveillance for anorectal cancer may help to improve cancer prognosis especially in patients with YO‐CDAAC, further studies to establish surveillance programs are needed in CDAAC.

## AUTHOR CONTRIBUTIONS

Guarantor of the article: Soichiro Ishihara, MD, PhD. Specific author contributions: YO is the corresponding author; contributed to the analysis, and the interpretation of data for the work and drafted the work.; YT contributed to the analysis and the interpretation of the data for the work and revised the work critically for important intellectual content. HI, MU, KF, and KO contributed to the acquisition of the data for the work and revised the work critically for important intellectual content. SI is the chief investigator of this study group and contributed to the design of the work, the acquisition and the interpretation of data for the work and revised the work critically for important intellectual content. TN, KS, and YA contributed to the conception of the work, the interpretation of the data for the work, and revised the work critically for important intellectual content.

## FUNDING INFORMATION

This work was supported by the Japanese Society of Cancer of the Colon and Rectum.

## CONFLICT OF INTEREST STATEMENT

The other authors declare no conflict of interest for this article. Yuji Toiyama is an editorial board member of *Annals of Gastroenterological Surgery*. The other authors declare no conflict of interest for this article.

## ETHICS STATEMENTS

Approval of the research protocol: This multicenter retrospective study was approved with the permission of the University of Tokyo Ethics Review Board (2019220NI‐(2)).

Informed Consent: N/A.

Registry and the Registration No. of the study/trial: N/A.

Animal Studies: N/A.

## Supporting information


Table S1.

Table S2.

Table S3.

Table S4.

Figure S1.

## References

[1] Lennard‐Jones JE , Melville DM , Morson BC , Ritchie JK , Williams CB . Precancer and cancer in extensive ulcerative colitis: findings among 401 patients over 22 years. Gut. 1990;31(7):800–806. 2370015 10.1136/gut.31.7.800 PMC1378540

[2] Munkholm P , Langholz E , Davidsen M , Binder V . Intestinal cancer risk and mortality in patients with Crohn's disease. Gastroenterology. 1993;105(6):1716–1723. 8253348 10.1016/0016-5085(93)91068-s

[3] Persson PG , Karlen P , Bernell O , Leijonmarck CE , Brostrom O , Ahlbom A , et al. Crohn's disease and cancer: a population‐based cohort study. Gastroenterology. 1994;107(6):1675–1679. 7958678 10.1016/0016-5085(94)90807-9

[4] Bernstein CN , Blanchard JF , Kliewer E , Wajda A . Cancer risk in patients with inflammatory bowel disease: a population‐based study. Cancer. 2001;91(4):854–862. 11241255 10.1002/1097-0142(20010215)91:4<854::aid-cncr1073>3.0.co;2-z

[5] Laukoetter MG , Mennigen R , Hannig CM , Osada N , Rijcken E , Vowinkel T , et al. Intestinal cancer risk in Crohn's disease: a meta‐analysis. J Gastrointest Surg. 2011;15(4):576–583. 21152994 10.1007/s11605-010-1402-9

[6] Uchino M , Ikeuchi H , Hata K , Minagawa T , Horio Y , Kuwahara R , et al. Intestinal cancer in patients with Crohn's disease: a systematic review and meta‐analysis. J Gastroenterol Hepatol. 2021;36(2):329–336. 32865278 10.1111/jgh.15229

[7] Higashi D , Katsuno H , Kimura H , Takahashi K , Ikeuchi H , Kono T , et al. Current state of and problems related to cancer of the intestinal tract associated with Crohn's disease in Japan. Anticancer Res. 2016;36(7):3761–3766. 27354651

[8] Olen O , Erichsen R , Sachs MC , Pedersen L , Halfvarson J , Askling J , et al. Colorectal cancer in Crohn's disease: a Scandinavian population‐based cohort study. Lancet Gastroenterol Hepatol. 2020;5(5):475–484. 32066530 10.1016/S2468-1253(20)30005-4

[9] Siegel RL , Torre LA , Soerjomataram I , Hayes RB , Bray F , Weber TK , et al. Global patterns and trends in colorectal cancer incidence in young adults. Gut. 2019;68(12):2179–2185. 31488504 10.1136/gutjnl-2019-319511

[10] Vuik FE , Nieuwenburg SA , Bardou M , Lansdorp‐Vogelaar I , Dinis‐Ribeiro M , Bento MJ , et al. Increasing incidence of colorectal cancer in young adults in Europe over the last 25 years. Gut. 2019;68(10):1820–1826. 31097539 10.1136/gutjnl-2018-317592 PMC6839794

[11] Mauri G , Sartore‐Bianchi A , Russo AG , Marsoni S , Bardelli A , Siena S . Early‐onset colorectal cancer in young individuals. Mol Oncol. 2019;13(2):109–131. 30520562 10.1002/1878-0261.12417 PMC6360363

[12] Cheng E , Blackburn HN , Ng K , Spiegelman D , Irwin ML , Ma X , et al. Analysis of survival among adults with early‐onset colorectal cancer in the National Cancer Database. JAMA Netw Open. 2021;4(6):e2112539. 34132794 10.1001/jamanetworkopen.2021.12539 PMC8209612

[13] Noguchi T , Ishihara S , Uchino M , Ikeuchi H , Okabayashi K , Futami K , et al. Clinical features and oncological outcomes of intestinal cancers associated with ulcerative colitis and Crohn's disease. J Gastroenterol. 2023;58(1):14–24. 36182971 10.1007/s00535-022-01927-y

[14] Proposed guidelines for the management of patients with Crohn's disease . Annual reports of Research Group of Intractable Inflammatory Bowel Disease subsidized by the Ministry of Health, Labor and Welfare of Japan. 2008. (in Japanese).

[15] Hirano Y , Futami K , Higashi D , Mikami K , Maekawa T . Anorectal cancer surveillance in Crohn's disease. J Anus Rectum Colon. 2018;2(4):145–154. 31559357 10.23922/jarc.2018-005 PMC6752139

[16] Ogino T , Mizushima T , Fujii M , Sekido Y , Eguchi H , Nezu R , et al. Crohn's disease‐associated anorectal cancer has a poor prognosis with high local recurrence: a subanalysis of the Nationwide Japanese study. Am J Gastroenterol. 2023;118:1626–1637. 36988310 10.14309/ajg.0000000000002269 PMC10453357

[17] Choi PM , Zelig MP . Similarity of colorectal cancer in Crohn's disease and ulcerative colitis: implications for carcinogenesis and prevention. Gut. 1994;35(7):950–954. 8063223 10.1136/gut.35.7.950 PMC1374843

[18] Ribeiro MB , Greenstein AJ , Sachar DB , Barth J , Balasubramanian S , Harpaz N , et al. Colorectal adenocarcinoma in Crohn's disease. Ann Surg. 1996;223(2):186–193. 8597513 10.1097/00000658-199602000-00011 PMC1235095

[19] Sasaki H , Ikeuchi H , Bando T , Hirose K , Hirata A , Chohno T , et al. Clinicopathological characteristics of cancer associated with Crohn's disease. Surg Today. 2017;47(1):35–41. 27094049 10.1007/s00595-016-1336-2 PMC5133282

[20] Benjelloun el B , Abkari M , Ousadden A , Ait Taleb K . Squamous cell carcinoma associated anal fistulas in Crohn's disease unique case report with literature review. J Crohns Colitis. 2013;7(6):e232–e235. 23069004 10.1016/j.crohns.2012.09.015

[21] Akimoto N , Ugai T , Zhong R , Hamada T , Fujiyoshi K , Giannakis M , et al. Rising incidence of early‐onset colorectal cancer – a call to action. Nat Rev Clin Oncol. 2021;18(4):230–243. 33219329 10.1038/s41571-020-00445-1 PMC7994182

[22] Chen FW , Sundaram V , Chew TA , Ladabaum U . Advanced‐stage colorectal cancer in persons younger than 50 years not associated with longer duration of symptoms or time to diagnosis. Clin Gastroenterol Hepatol. 2017;15(5):728–737.e3. 27856366 10.1016/j.cgh.2016.10.038 PMC5401776

[23] Siegel RL , Jakubowski CD , Fedewa SA , Davis A , Azad NS . Colorectal cancer in the young: epidemiology, prevention, management. Am Soc Clin Oncol Educ Book. 2020;40:1–14. 10.1200/EDBK_279901 32315236

[24] Hata K , Ishihara S , Watanabe T . Successful surveillance colonoscopy for patients with ulcerative colitis after Ileorectal anastomosis. J Crohns Colitis. 2015;9(10):937–938. 26188348 10.1093/ecco-jcc/jjv124

[25] Hata K , Anzai H , Ikeuchi H , Futami K , Fukushima K , Sugita A , et al. Surveillance colonoscopy for ulcerative colitis‐associated colorectal cancer offers better overall survival in real‐world surgically resected cases. Am J Gastroenterol. 2019;114(3):483–489. 30747769 10.14309/ajg.0000000000000117

[26] Itzkowitz SH , Present DH . Crohn's, colitis Foundation of America Colon Cancer in IBDSG, consensus conference: colorectal cancer screening and surveillance in inflammatory bowel disease. Inflamm Bowel Dis. 2005;11(3):314–321. 15735438 10.1097/01.mib.0000160811.76729.d5

[27] Cairns SR , Scholefield JH , Steele RJ , Dunlop MG , Thomas HJ , Evans GD , et al. Guidelines for colorectal cancer screening and surveillance in moderate and high risk groups (update from 2002). Gut. 2010;59(5):666–689. 20427401 10.1136/gut.2009.179804

[28] Torres J , Caprioli F , Katsanos KH , Lobaton T , Micic D , Zeroncio M , et al. Predicting outcomes to optimize disease management in inflammatory bowel diseases. J Crohns Colitis. 2016;10(12):1385–1394. 27282402 10.1093/ecco-jcc/jjw116 PMC5174730

[29] Kotsafti A , Scarpa M , Angriman I , Castagliuolo I , Caruso A . Fistula‐related cancer in Crohn's disease: a systematic review. Cancers (Basel). 2021;13(6):1445. 33809997 10.3390/cancers13061445 PMC8005214

