# Supplementary material for: Possible poor prognosis in younger‐onset Crohn's disease‐associated anorectal cancer: A subanalysis of the Nationwide Japanese study
Source: Ann Gastroenterol Surg. 2024 Jan 27;8(4):620–30. doi: 10.1002/ags3.12773 (PMC11216786; doi:10.1002/ags3.12773)
Supplement: Supplementary file 1 — Table S1. [file AGS3-8-620-s004.pptx]

## Slide 1
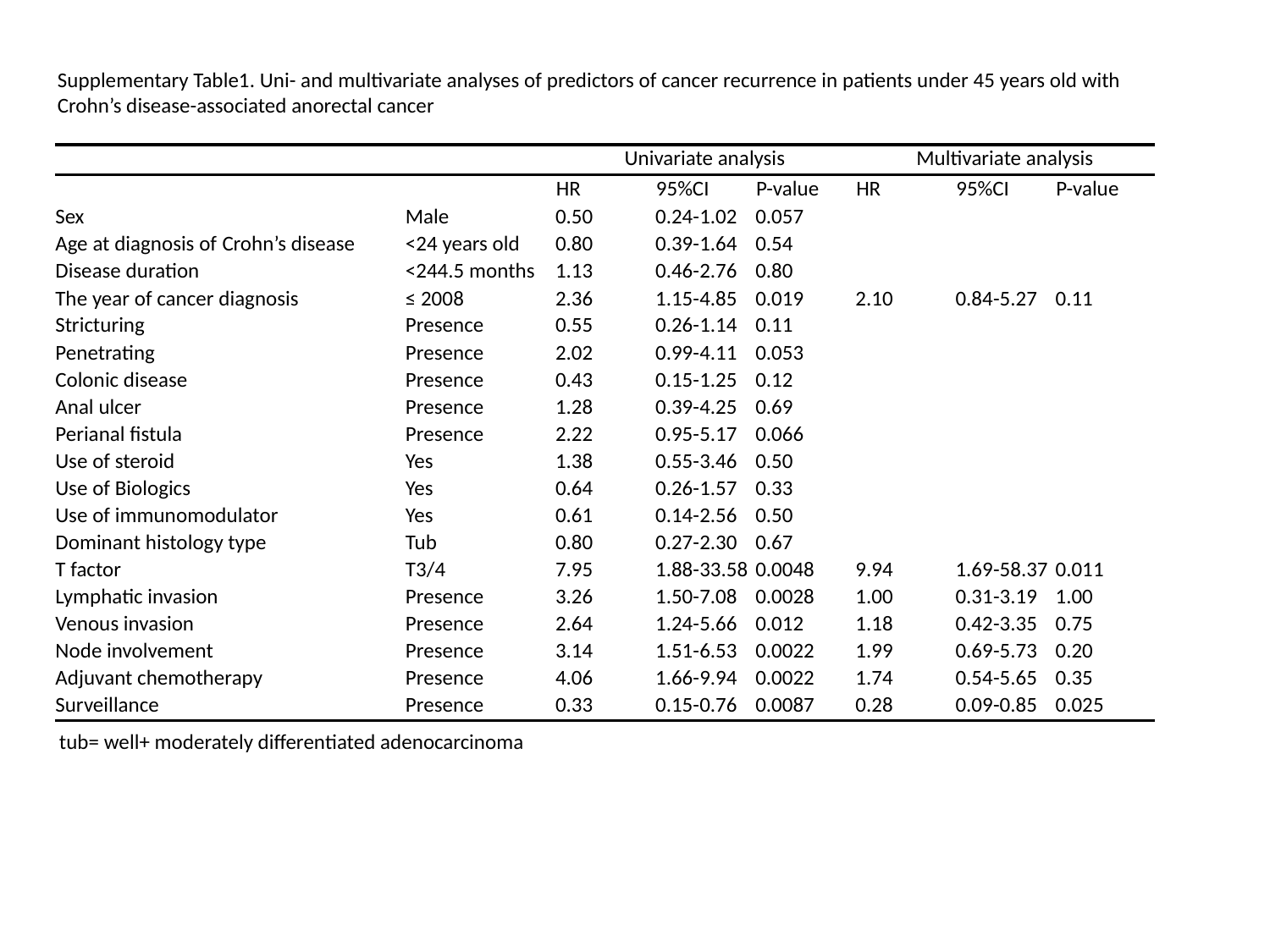

Supplementary Table1. Uni- and multivariate analyses of predictors of cancer recurrence in patients under 45 years old with Crohn’s disease-associated anorectal cancer
| | | Univariate analysis | | | Multivariate analysis | | |
| --- | --- | --- | --- | --- | --- | --- | --- |
| | | HR | 95%CI | P-value | HR | 95%CI | P-value |
| Sex | Male | 0.50 | 0.24-1.02 | 0.057 | | | |
| Age at diagnosis of Crohn’s disease | <24 years old | 0.80 | 0.39-1.64 | 0.54 | | | |
| Disease duration | <244.5 months | 1.13 | 0.46-2.76 | 0.80 | | | |
| The year of cancer diagnosis | ≤ 2008 | 2.36 | 1.15-4.85 | 0.019 | 2.10 | 0.84-5.27 | 0.11 |
| Stricturing | Presence | 0.55 | 0.26-1.14 | 0.11 | | | |
| Penetrating | Presence | 2.02 | 0.99-4.11 | 0.053 | | | |
| Colonic disease | Presence | 0.43 | 0.15-1.25 | 0.12 | | | |
| Anal ulcer | Presence | 1.28 | 0.39-4.25 | 0.69 | | | |
| Perianal fistula | Presence | 2.22 | 0.95-5.17 | 0.066 | | | |
| Use of steroid | Yes | 1.38 | 0.55-3.46 | 0.50 | | | |
| Use of Biologics | Yes | 0.64 | 0.26-1.57 | 0.33 | | | |
| Use of immunomodulator | Yes | 0.61 | 0.14-2.56 | 0.50 | | | |
| Dominant histology type | Tub | 0.80 | 0.27-2.30 | 0.67 | | | |
| T factor | T3/4 | 7.95 | 1.88-33.58 | 0.0048 | 9.94 | 1.69-58.37 | 0.011 |
| Lymphatic invasion | Presence | 3.26 | 1.50-7.08 | 0.0028 | 1.00 | 0.31-3.19 | 1.00 |
| Venous invasion | Presence | 2.64 | 1.24-5.66 | 0.012 | 1.18 | 0.42-3.35 | 0.75 |
| Node involvement | Presence | 3.14 | 1.51-6.53 | 0.0022 | 1.99 | 0.69-5.73 | 0.20 |
| Adjuvant chemotherapy | Presence | 4.06 | 1.66-9.94 | 0.0022 | 1.74 | 0.54-5.65 | 0.35 |
| Surveillance | Presence | 0.33 | 0.15-0.76 | 0.0087 | 0.28 | 0.09-0.85 | 0.025 |
tub= well+ moderately differentiated adenocarcinoma
