# Supplementary material for: Possible poor prognosis in younger‐onset Crohn's disease‐associated anorectal cancer: A subanalysis of the Nationwide Japanese study
Source: Ann Gastroenterol Surg. 2024 Jan 27;8(4):620–30. doi: 10.1002/ags3.12773 (PMC11216786; doi:10.1002/ags3.12773)
Supplement: Supplementary file 2 — Table S2. [file AGS3-8-620-s002.pptx]

## Slide 1
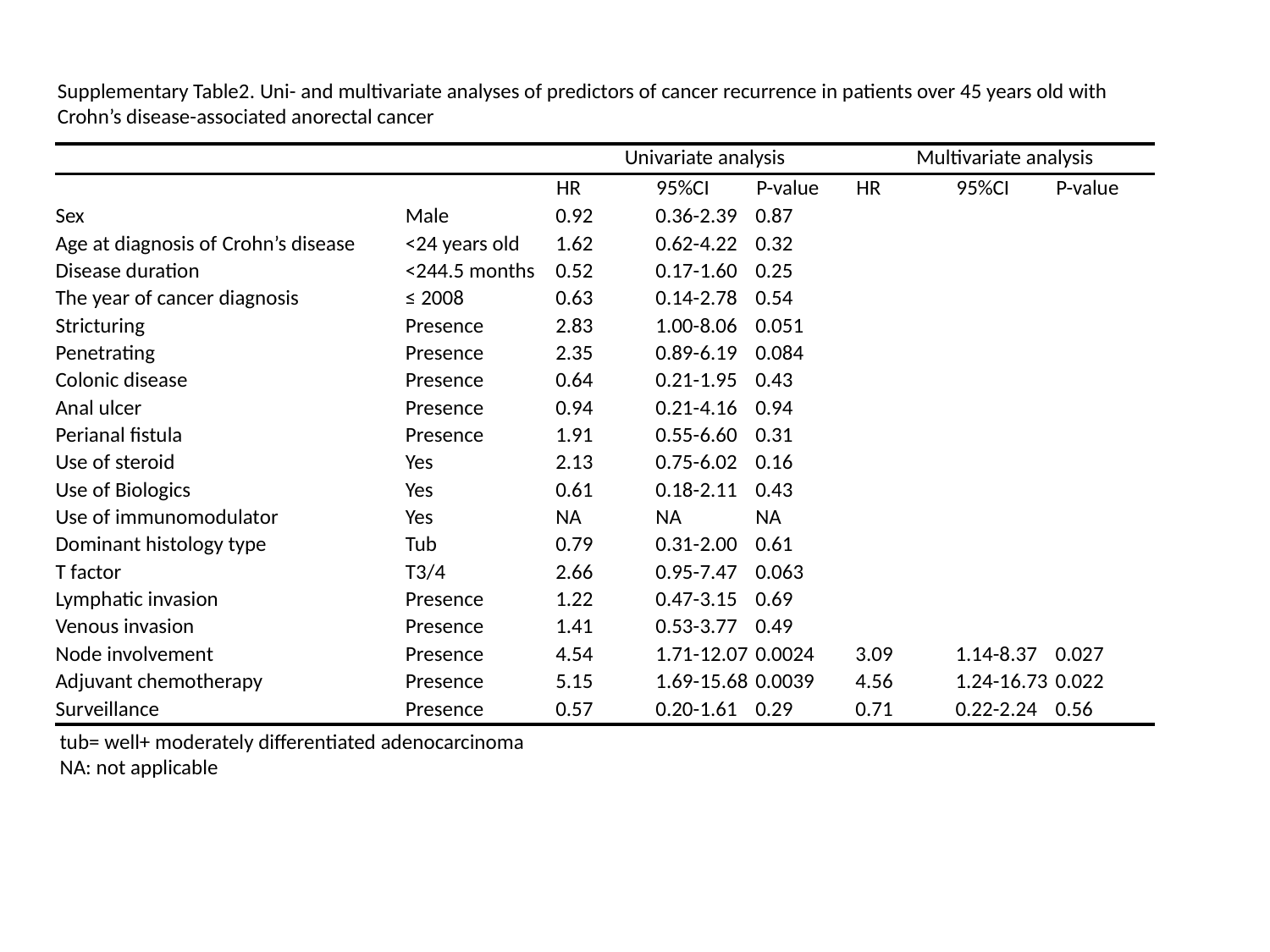

Supplementary Table2. Uni- and multivariate analyses of predictors of cancer recurrence in patients over 45 years old with Crohn’s disease-associated anorectal cancer
| | | Univariate analysis | | | Multivariate analysis | | |
| --- | --- | --- | --- | --- | --- | --- | --- |
| | | HR | 95%CI | P-value | HR | 95%CI | P-value |
| Sex | Male | 0.92 | 0.36-2.39 | 0.87 | | | |
| Age at diagnosis of Crohn’s disease | <24 years old | 1.62 | 0.62-4.22 | 0.32 | | | |
| Disease duration | <244.5 months | 0.52 | 0.17-1.60 | 0.25 | | | |
| The year of cancer diagnosis | ≤ 2008 | 0.63 | 0.14-2.78 | 0.54 | | | |
| Stricturing | Presence | 2.83 | 1.00-8.06 | 0.051 | | | |
| Penetrating | Presence | 2.35 | 0.89-6.19 | 0.084 | | | |
| Colonic disease | Presence | 0.64 | 0.21-1.95 | 0.43 | | | |
| Anal ulcer | Presence | 0.94 | 0.21-4.16 | 0.94 | | | |
| Perianal fistula | Presence | 1.91 | 0.55-6.60 | 0.31 | | | |
| Use of steroid | Yes | 2.13 | 0.75-6.02 | 0.16 | | | |
| Use of Biologics | Yes | 0.61 | 0.18-2.11 | 0.43 | | | |
| Use of immunomodulator | Yes | NA | NA | NA | | | |
| Dominant histology type | Tub | 0.79 | 0.31-2.00 | 0.61 | | | |
| T factor | T3/4 | 2.66 | 0.95-7.47 | 0.063 | | | |
| Lymphatic invasion | Presence | 1.22 | 0.47-3.15 | 0.69 | | | |
| Venous invasion | Presence | 1.41 | 0.53-3.77 | 0.49 | | | |
| Node involvement | Presence | 4.54 | 1.71-12.07 | 0.0024 | 3.09 | 1.14-8.37 | 0.027 |
| Adjuvant chemotherapy | Presence | 5.15 | 1.69-15.68 | 0.0039 | 4.56 | 1.24-16.73 | 0.022 |
| Surveillance | Presence | 0.57 | 0.20-1.61 | 0.29 | 0.71 | 0.22-2.24 | 0.56 |
tub= well+ moderately differentiated adenocarcinoma
NA: not applicable
