# Supplementary material for: Possible poor prognosis in younger‐onset Crohn's disease‐associated anorectal cancer: A subanalysis of the Nationwide Japanese study
Source: Ann Gastroenterol Surg. 2024 Jan 27;8(4):620–30. doi: 10.1002/ags3.12773 (PMC11216786; doi:10.1002/ags3.12773)
Supplement: Supplementary file 3 — Table S3. [file AGS3-8-620-s001.pptx]

## Slide 1
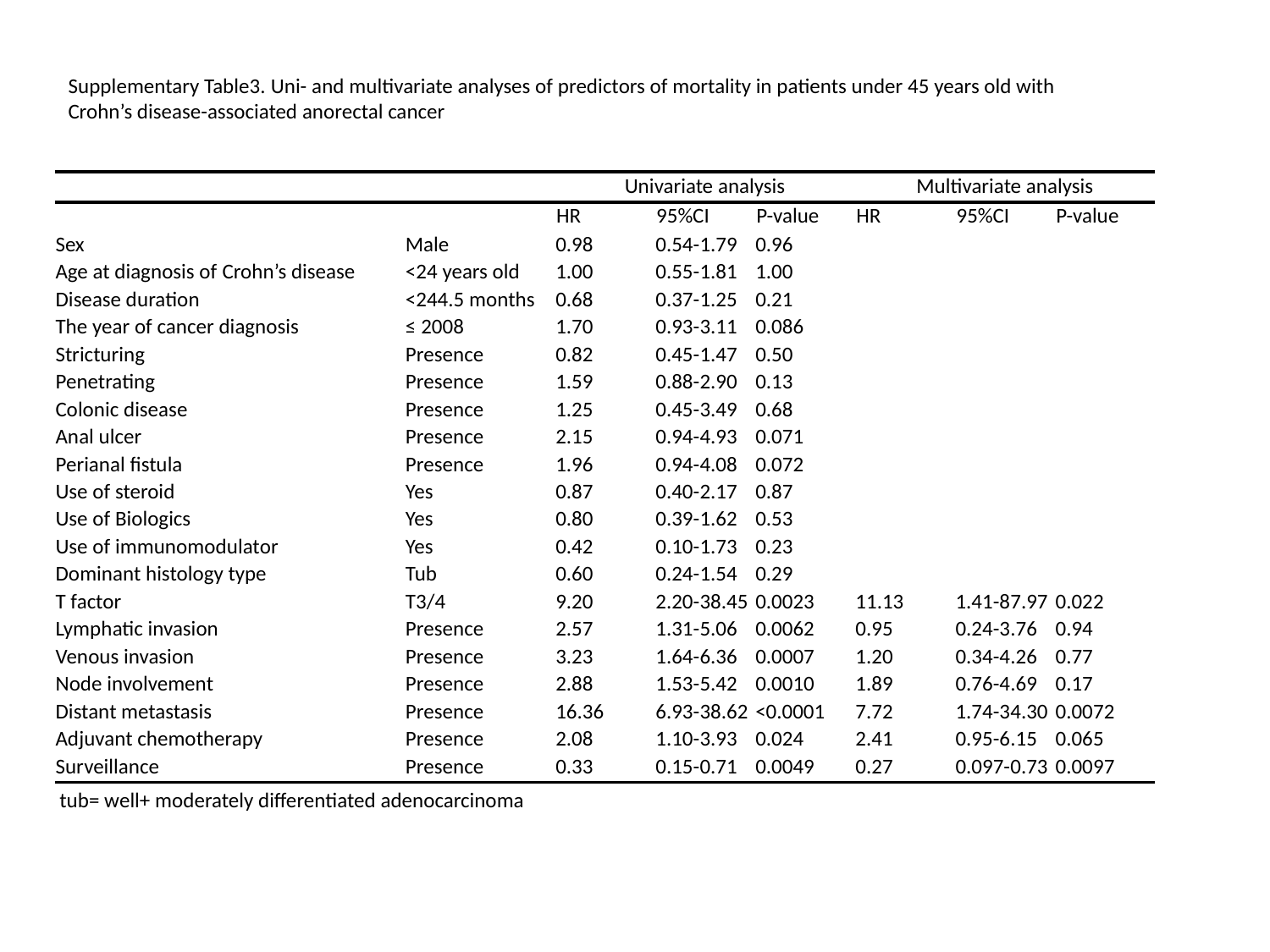

Supplementary Table3. Uni- and multivariate analyses of predictors of mortality in patients under 45 years old with Crohn’s disease-associated anorectal cancer
| | | Univariate analysis | | | Multivariate analysis | | |
| --- | --- | --- | --- | --- | --- | --- | --- |
| | | HR | 95%CI | P-value | HR | 95%CI | P-value |
| Sex | Male | 0.98 | 0.54-1.79 | 0.96 | | | |
| Age at diagnosis of Crohn’s disease | <24 years old | 1.00 | 0.55-1.81 | 1.00 | | | |
| Disease duration | <244.5 months | 0.68 | 0.37-1.25 | 0.21 | | | |
| The year of cancer diagnosis | ≤ 2008 | 1.70 | 0.93-3.11 | 0.086 | | | |
| Stricturing | Presence | 0.82 | 0.45-1.47 | 0.50 | | | |
| Penetrating | Presence | 1.59 | 0.88-2.90 | 0.13 | | | |
| Colonic disease | Presence | 1.25 | 0.45-3.49 | 0.68 | | | |
| Anal ulcer | Presence | 2.15 | 0.94-4.93 | 0.071 | | | |
| Perianal fistula | Presence | 1.96 | 0.94-4.08 | 0.072 | | | |
| Use of steroid | Yes | 0.87 | 0.40-2.17 | 0.87 | | | |
| Use of Biologics | Yes | 0.80 | 0.39-1.62 | 0.53 | | | |
| Use of immunomodulator | Yes | 0.42 | 0.10-1.73 | 0.23 | | | |
| Dominant histology type | Tub | 0.60 | 0.24-1.54 | 0.29 | | | |
| T factor | T3/4 | 9.20 | 2.20-38.45 | 0.0023 | 11.13 | 1.41-87.97 | 0.022 |
| Lymphatic invasion | Presence | 2.57 | 1.31-5.06 | 0.0062 | 0.95 | 0.24-3.76 | 0.94 |
| Venous invasion | Presence | 3.23 | 1.64-6.36 | 0.0007 | 1.20 | 0.34-4.26 | 0.77 |
| Node involvement | Presence | 2.88 | 1.53-5.42 | 0.0010 | 1.89 | 0.76-4.69 | 0.17 |
| Distant metastasis | Presence | 16.36 | 6.93-38.62 | <0.0001 | 7.72 | 1.74-34.30 | 0.0072 |
| Adjuvant chemotherapy | Presence | 2.08 | 1.10-3.93 | 0.024 | 2.41 | 0.95-6.15 | 0.065 |
| Surveillance | Presence | 0.33 | 0.15-0.71 | 0.0049 | 0.27 | 0.097-0.73 | 0.0097 |
tub= well+ moderately differentiated adenocarcinoma
