# Supplementary material for: Possible poor prognosis in younger‐onset Crohn's disease‐associated anorectal cancer: A subanalysis of the Nationwide Japanese study
Source: Ann Gastroenterol Surg. 2024 Jan 27;8(4):620–30. doi: 10.1002/ags3.12773 (PMC11216786; doi:10.1002/ags3.12773)
Supplement: Supplementary file 4 — Table S4. [file AGS3-8-620-s003.pptx]

## Slide 1
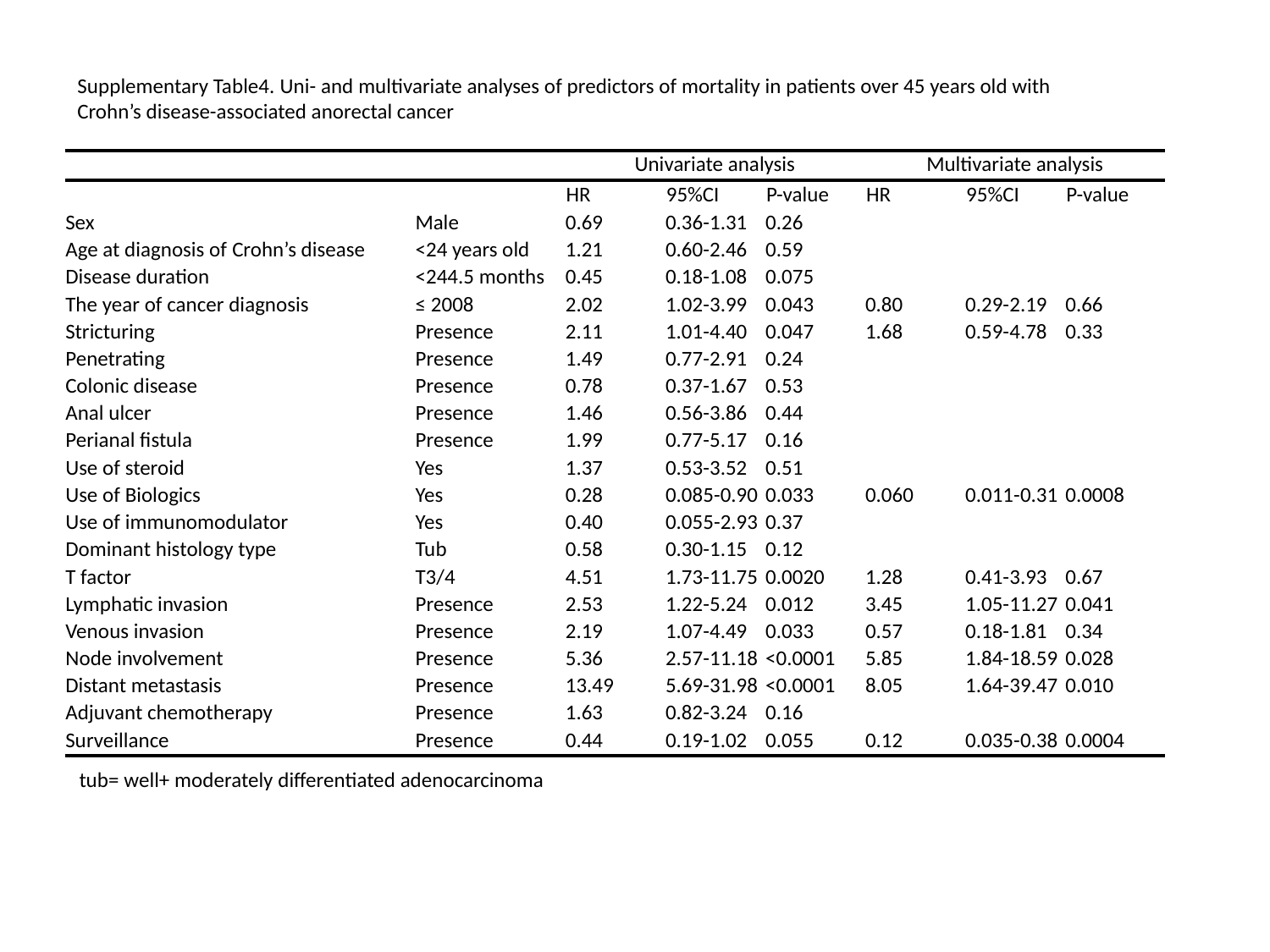

Supplementary Table4. Uni- and multivariate analyses of predictors of mortality in patients over 45 years old with Crohn’s disease-associated anorectal cancer
| | | Univariate analysis | | | Multivariate analysis | | |
| --- | --- | --- | --- | --- | --- | --- | --- |
| | | HR | 95%CI | P-value | HR | 95%CI | P-value |
| Sex | Male | 0.69 | 0.36-1.31 | 0.26 | | | |
| Age at diagnosis of Crohn’s disease | <24 years old | 1.21 | 0.60-2.46 | 0.59 | | | |
| Disease duration | <244.5 months | 0.45 | 0.18-1.08 | 0.075 | | | |
| The year of cancer diagnosis | ≤ 2008 | 2.02 | 1.02-3.99 | 0.043 | 0.80 | 0.29-2.19 | 0.66 |
| Stricturing | Presence | 2.11 | 1.01-4.40 | 0.047 | 1.68 | 0.59-4.78 | 0.33 |
| Penetrating | Presence | 1.49 | 0.77-2.91 | 0.24 | | | |
| Colonic disease | Presence | 0.78 | 0.37-1.67 | 0.53 | | | |
| Anal ulcer | Presence | 1.46 | 0.56-3.86 | 0.44 | | | |
| Perianal fistula | Presence | 1.99 | 0.77-5.17 | 0.16 | | | |
| Use of steroid | Yes | 1.37 | 0.53-3.52 | 0.51 | | | |
| Use of Biologics | Yes | 0.28 | 0.085-0.90 | 0.033 | 0.060 | 0.011-0.31 | 0.0008 |
| Use of immunomodulator | Yes | 0.40 | 0.055-2.93 | 0.37 | | | |
| Dominant histology type | Tub | 0.58 | 0.30-1.15 | 0.12 | | | |
| T factor | T3/4 | 4.51 | 1.73-11.75 | 0.0020 | 1.28 | 0.41-3.93 | 0.67 |
| Lymphatic invasion | Presence | 2.53 | 1.22-5.24 | 0.012 | 3.45 | 1.05-11.27 | 0.041 |
| Venous invasion | Presence | 2.19 | 1.07-4.49 | 0.033 | 0.57 | 0.18-1.81 | 0.34 |
| Node involvement | Presence | 5.36 | 2.57-11.18 | <0.0001 | 5.85 | 1.84-18.59 | 0.028 |
| Distant metastasis | Presence | 13.49 | 5.69-31.98 | <0.0001 | 8.05 | 1.64-39.47 | 0.010 |
| Adjuvant chemotherapy | Presence | 1.63 | 0.82-3.24 | 0.16 | | | |
| Surveillance | Presence | 0.44 | 0.19-1.02 | 0.055 | 0.12 | 0.035-0.38 | 0.0004 |
tub= well+ moderately differentiated adenocarcinoma
