# Supplementary material for: Possible poor prognosis in younger‐onset Crohn's disease‐associated anorectal cancer: A subanalysis of the Nationwide Japanese study
Source: Ann Gastroenterol Surg. 2024 Jan 27;8(4):620–30. doi: 10.1002/ags3.12773 (PMC11216786; doi:10.1002/ags3.12773)
Supplement: Supplementary file 5 — Figure S1. [file AGS3-8-620-s005.pptx]

## Slide 1
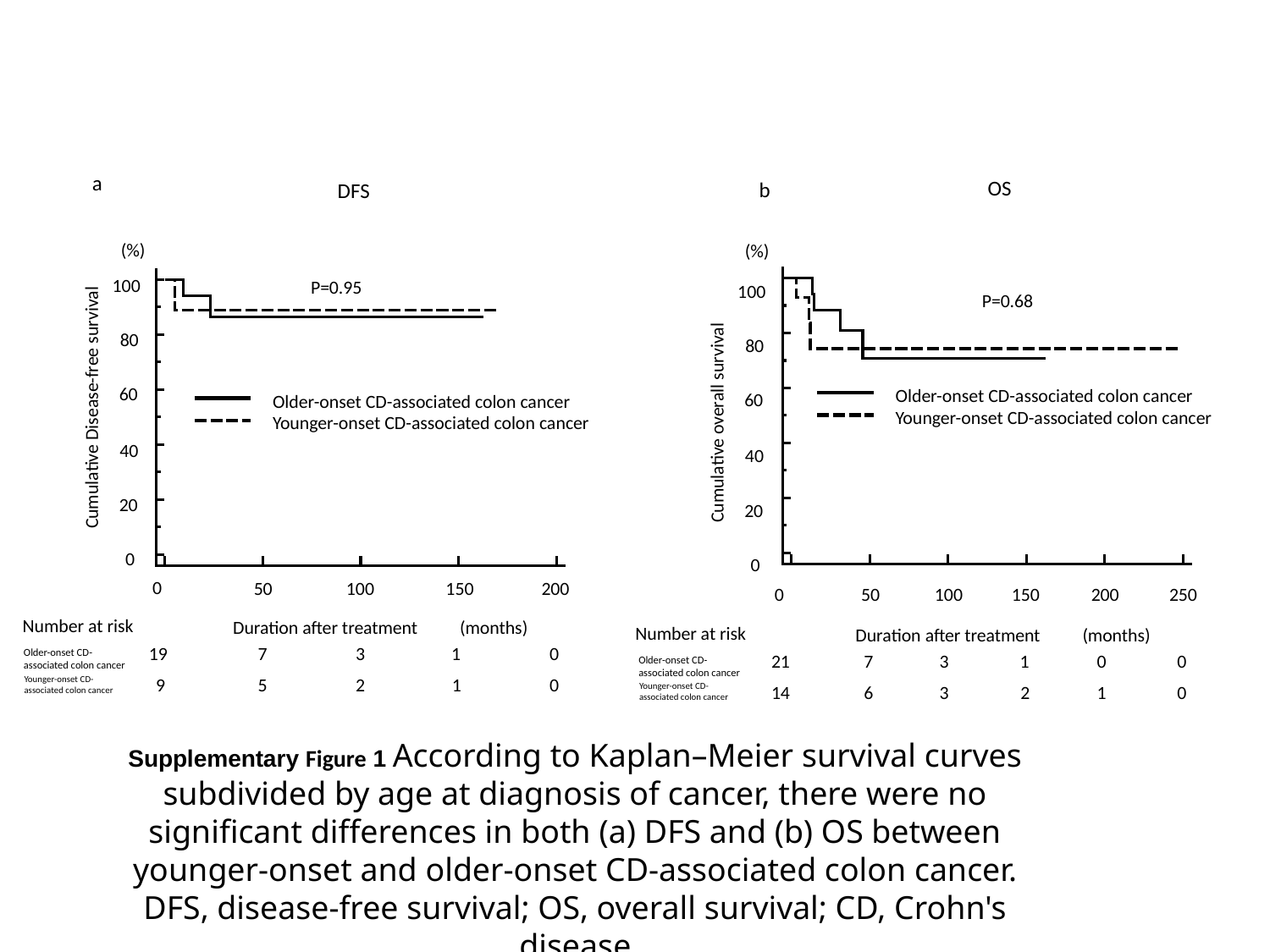

a
OS
b
 DFS
(%)
(%)
P=0.95
100
100
P=0.68
80
80
Older-onset CD-associated colon cancer
Younger-onset CD-associated colon cancer
60
Older-onset CD-associated colon cancer
Younger-onset CD-associated colon cancer
60
Cumulative Disease-free survival
Cumulative overall survival
40
40
20
20
0
0
0
50
100
150
200
0
50
100
150
200
250
Duration after treatment
(months)
Number at risk
Duration after treatment
(months)
Number at risk
Older-onset CD-associated colon cancer
19
7
3
1
0
Older-onset CD-associated colon cancer
21
7
3
1
0
0
Younger-onset CD-associated colon cancer
9
5
2
1
0
Younger-onset CD-associated colon cancer
14
6
3
2
1
0
Supplementary Figure 1 According to Kaplan–Meier survival curves subdivided by age at diagnosis of cancer, there were no significant differences in both (a) DFS and (b) OS between younger-onset and older-onset CD-associated colon cancer. DFS, disease-free survival; OS, overall survival; CD, Crohn's disease
